# Alterations in Serum-Free Amino Acid Profiles in Childhood Asthma

**DOI:** 10.3390/ijerph17134758

**Published:** 2020-07-02

**Authors:** Joanna Matysiak, Agnieszka Klupczynska, Kacper Packi, Anna Mackowiak-Jakubowska, Anna Bręborowicz, Olga Pawlicka, Katarzyna Olejniczak, Zenon J. Kokot, Jan Matysiak

**Affiliations:** 1 Faculty of Health Sciences, The President Stanisław Wojciechowski State University of Applied Sciences in Kalisz, 62-800 Kalisz, Poland; zkokot@ump.edu.pl; 2 Department of Inorganic and Analytical Chemistry, Poznan University of Medical Sciences, 60 -780 Poznan, Poland; aklupczynska@ump.edu.pl (A.K.); kacperpacki1@wp.pl (K.P.); amackowiak@gmail.com (A.M.-J.); olga_szklanny@interia.pl (O.P.); jmatysiak@ump.edu.pl (J.M.); 3 Department of Pulmonology, Pediatric Allergy and Clinical Immunology, Poznan University of Medical Sciences, 60-572 Poznan, Poland; abreborowicz@wp.pl (A.B.); kasiatomeko@gmail.com (K.O.)

**Keywords:** metabolomics, metabolites, amino acids, biomarkers, asthma, diagnosis, children

## Abstract

Asthma often begins in childhood, although making an early diagnosis is difficult. Clinical manifestations, the exclusion of other causes of bronchial obstruction, and responsiveness to anti-inflammatory therapy are the main tool of diagnosis. However, novel, precise, and functional biochemical markers are needed in the differentiation of asthma phenotypes, endotypes, and creating personalized therapy. The aim of the study was to search for metabolomic-based asthma biomarkers among free amino acids (AAs). A wide panel of serum-free AAs in asthmatic children, covering both proteinogenic and non-proteinogenic AAs, were analyzed. The examination included two groups of individuals between 3 and 18 years old: asthmatic children and the control group consisted of children with neither asthma nor allergies. High-performance liquid chromatography combined with tandem mass spectrometry (LC-MS/MS technique) was used for AA measurements. The data were analyzed by applying uni- and multivariate statistical tests. The obtained results indicate the decreased serum concentration of taurine, L-valine, DL-β-aminoisobutyric acid, and increased levels of ƴ-amino-n-butyric acid and L-arginine in asthmatic children when compared to controls. The altered concentration of these AAs can testify to their role in the pathogenesis of childhood asthma. The authors’ results should contribute to the future introduction of new diagnostic markers into clinical practice.

## 1. Introduction

Asthma is a serious problem in contemporary medicine. It is a chronic illness, caused by both genetic and environmental factors and characterized by the inflammation of the lower airways. Inflammation leads to oedema and the infiltration of the bronchial mucosa, bronchospasm, and an excessive mucus secretion, resulting in reversible airway obstruction. However, in a significant percentage of patients, chronic inflammation leads to the remodeling of the bronchial walls and fixed airflow limitation. The highest incidence rates of asthma are recorded in developed countries [1]. The recent global epidemiological report has shown that nowadays this ailment is affecting more than 300 million people all over the world. In many patients, asthma begins in childhood [2]. Recurrent wheezing episodes triggered by viral infection are the most common manifestation of asthma in infancy and preschool children. However, the nonspecific symptoms, such as wheezing, cough, and chest tightness, make it difficult to distinguish asthma from other diseases [3,4]. An atopic background predisposes to asthma development but is not an essential condition. Objective tests supporting preschool asthma diagnoses are not commonly available and clinical criteria remain a major diagnostic tool in clinical practice. There are significant differences between childhood and adulthood asthma, concerning immunology, histopathology, and clinical manifestations.

Many researchers have tried to identify specific biological markers of childhood asthma that would aid in the diagnosis of this disorder. Finding such compounds is extremely important because accurate diagnosis and optimal treatment play a crucial role in the proper functioning of asthmatic children and can greatly improve their quality of life.

One of the disciplines being used to discover new biomarkers of various diseases and to identify biochemical pathways involved in their pathogenesis is metabolomics. This latest of the so-called omic techniques is focused on low-molecular-weight intermediates and the products of metabolism, such as amino acids, fatty acids, carbohydrates, or nucleotides. The composition of the metabolome is determined by the current condition of the organism, which is affected by external and internal factors, including pathology, applied therapy, or diet [5,6]. It was found that changes in the human metabolome precede clinical symptoms’ occurrence [7]. Therefore, the metabolomic approach offers a powerful tool in the development of new diagnostic tests, including respiratory tract diseases.

In metabolomic research, two strategies are used: targeted and non-targeted analysis. A targeted approach is used for the precise quantitative analysis of a limited number of metabolites with known biochemical properties. By using highly sensitive and selective analytical techniques, a certain group of metabolites can be detected and quantified, even if they are present in very low concentrations. A non-targeted analysis, also called metabolite fingerprinting, enables the classification of the tested biological material on the basis of the complete metabolic profile, rather than individual substances. The non-targeted strategy aims to structurally detect diverse compounds present in the metabolome, however, without quantitative data [5,6,8,9].

Metabolomics has provided potential biomarkers of asthma belonging to different groups of compounds that take part in various biological pathways. Metabolomics was applied to urine [10,11], plasma/serum [12,13,14,15,16,17], and exhaled breath condensate (EBC) samples [18]. By applying the metabolomic profiling of urine, the significant differences in the concentrations of phenylalanine, glycolic acid, 2-oxoglutarate, threonine, and phenylacetic acid were observed between asthmatic children and healthy ones [10]. In 2013, another research group explicated that the serum of asthmatics is characterized by increased levels of glutamine, histidine, and methionine and by decreased levels of acetate, arginine, choline, formate, glucose, methanol, and O-phosphocholine [15]. Abnormalities were also found in the serum levels of lysophosphatidylcholines and phosphatidylcholines [16]. In 2013, the metabolome of breath condensate was also tested. That study indicated that compounds related to adenosine, retinoic acid, and vitamin D may be used in the profiling of different asthma phenotypes [18].

Free amino acids (AAs) represent a particularly interesting group of metabolites worth examining among asthmatic children [19,20]. Amino acids can be divided into proteinogenic and non-proteinogenic. Proteinogenic AAs are incorporated into proteins during translation and encompass twenty-two AAs. Non-proteinogenic AAs are not naturally encoded or found in the genetic code of any organism, but most of them also play important functions in the organism (i.e., carnitine, creatine, ornithine, taurine, hydroxyproline, hydroxylysine). Non-proteinogenic amino acids often occur as intermediates in the metabolic pathways for standard AAs [21,22]. Abnormalities in free AA levels in body fluids have been reported in various disorders, i.a., liver diseases [23], chronic renal failure [24,25], neoplastic diseases [26,27,28,29,30], diabetes mellitus [31,32], and obesity [31,33].

A variety of methods allow the determination of AAs in biological samples. The most commonly used analytical techniques include high-performance liquid chromatography (HPLC), gas chromatography combined with mass spectrometry (GC-MS), and high-performance liquid chromatography coupled with mass spectrometry (LC-MS/MS). The ideal technique for determining the amino acid profile should be characterized by: high resolution, selectivity, the ability to determine the largest amount of amino acids in one analytical cycle, short duration, high sensitivity, repeatability, and low cost of analysis. Poschke et al. [34] used the HPLC technique to determine free AAs in blood serum. The method used by the researchers allowed the simultaneous determination of 15 AAs. The duration of a single analytical cycle was a total of 31 min (28 min analysis and 3 min column regeneration). In addition, before each analytical cycle, a derivatization reaction was necessary. Shi et al. also used the LC technique [35]. This method enabled the determination of 19 AA concentrations. The duration of a single cycle was 30 min. The analysis was preceded by the column equilibration, lasting 30 min. Deng et al. [36] used gas chromatography coupled with mass spectrometry to determine free AAs in the blood of newborns. The technique enabled the simultaneous determination of five AAs and required two-step derivatization (esterification and acylation). The duration of a single analytical run was 15 min. All described methods were characterized by high resolution, repeatability, accuracy, and selectivity [35,36]. We applied high-performance liquid chromatography coupled with mass spectrometry (LC-MS/MS) that, in addition to mentioned advantages, allows the determination of the widest spectrum of free amino acids (42 analytes. LC-MS/MS is also characterized by a short duration of a single analysis-18 min-which is particularly important when determining a large number of samples, as well as the ability to analyze body fluids of various origins.

The examination of free serum AAs would contribute not only to estimating their diagnostic utility in asthmatic children but will also contribute to broadening the knowledge about the mechanisms of this disorder. Scientists attempted several times to find the relation between selected AA levels and asthma occurrence [14,37]. However, the research projects which have been conducted so far failed to clearly answer the question of the impact of asthma on the profile of free AAs in human blood. Therefore, it is necessary to analyze the broad profile of free AAs in asthmatics and compare the levels of these metabolites with healthy subjects. Children are a particularly interesting research group, due to the different course of the disease in comparison with adults. To date, only a few metabolomic studies have been devoted to the pediatric population [10,11,17,18], and none of them showed a wide range of free AAs in serum. The current research aimed at applying the targeted metabolomic approach in order to find the differences with potential diagnostic significance in asthma. Previously conducted serum research has focused mainly on the altered levels of arginine and arginase activity [12]. This is the first study that presents the analysis of a wide panel of free AAs in asthmatic children, covering both proteinogenic and non-proteinogenic AAs.

## 2. Methods

### 2.1. Chemicals and Reagents

The analysis of free AAs was performed using an aTRAQ kit (Sciex, Framingham, MA, USA) that allows for quantifying of the 42 free amino acids (proteinogenic and non-proteinogenic) in a range of biological fluids. Deionized water was obtained from a Simplicity UV (Merck Millipore, Darmstadt, Germany) purifying system. HPLC gradient grade methanol was supplied by J.T. Baker (Center Valley, PA, USA).

### 2.2. Patients

The study was conducted involving two groups of subjects between 3 and 18 years of age: asthmatic children (*n* = 13) and the control group, which consisted of children with neither asthma nor allergies (*n* = 17). Children were recruited from the Department of Pediatric Pneumonology, Allergology and Clinical Immunology, K. Jonscher Clinical Hospital, Poznan University of Medical Sciences, after the written consent of their parents. Thirty serum samples were the test material, and they were stored at −80 °C until analysis. The study was approved by the Local Ethical Committee of Poznan University of Medical Sciences, Poland (Decision No. 530/12), and was consistent with the requirements of the Helsinki declaration.

### 2.3. Sample Collection and Preparation

Blood samples from both groups (asthmatic children and control group) were collected, processed, and stored in the same way. Blood was collected during medical examination into tubes with a clotting activator (S-Monovette system, Sarstedt, Nümbrecht, Germany). Then, sera were obtained by centrifugation at 300 × 10 RPM for 20 min. The sera were aliquoted and stored at −80 °C until analysis. All samples used in the study were analyzed on the first freeze-thaw cycle.

For AA determination in the collected sera, an aTRAQ reagent kit was used. The reliability of the method was confirmed in the literature [38,39,40]. The advantages of the aTRAQ methodology include a low sample volume required for analysis, a broad range of analytes (Table S1), and the use of labeled internal standards for each amino acid, which provide accurate quantitative results. The first step of the preparation procedure was the thawing of samples and transferring 40 μL of serum into Eppendorf tubes. Then 10 μL of 10% sulfosalicylic acid were added to precipitate proteins. The samples were mixed and centrifuged (2 min; 10,000× *g*). Then 10 μL of the supernatant were transferred to a clean tube and mixed with 40 μL of borate buffer (pH = 8.5). After mixing and centrifuging, 10 μL of the solution were transferred to a clean tube and 5 μL of labeling reagent solution (aTRAQ Reagent Δ8) were added. The samples were incubated for 30 min at room temperature. After that, 5 μL of hydroxylamine were added to stop the labeling reaction and the samples were incubated at room temperature for 15 min. Then samples were mixed with 32 μL of the internal standard solution. The tube contents were concentrated to a volume of approximately 20 μL using a vacuum concentrator (miVac Duo Concentrator, Genevac, Stone Ridge, NY, USA). Then samples were mixed with 20 μL of water and transferred to an autosampler vial.

### 2.4. LC-MS/MS Instrumentation

The measurements of AA concentrations were conducted using liquid chromatography-tandem mass spectrometry (LC-MS/MS) and a fully validated, highly selective method [38,39]. The applied method offers a valid alternative to the most conventional method of AA quantification that employs ion exchange chromatography (IEC) followed by post column ninhydrin derivatization and UV detection [41]. The LC-MS/MS method offers several advantages compared to the IEC method: decreased run time, a high amount of analytes quantified in one analytical run, low limits of quantification, and superior specificity via the use of the scheduled multiple reaction monitoring (sMRM) mode [38,39,40]. The analyses were performed on the 1260 Infinity HPLC system (Agilent Technologies, Santa Clara, CA, USA) coupled with a 4000 QTRAP triple quadrupole mass spectrometer (Sciex, Framingham, MA, USA) with an electrospray ionization (ESI) source. The chromatographic separation was carried out using a C18 column (4.6 mm × 150 mm, 5 µm) (Sciex, Framingham, MA, USA) with a flow rate of 0.8 mL/min. The eluent A was composed of water with 0.1% formic acid and 0.01% heptafluorobutyric acid and the eluent B contained methanol with 0.1% formic acid and 0.01% heptafluorobutyric acid. The gradient elution program was as follows: 0 min, 2% B; 0–6 min linear from 2 to 40% B; 6–10 min, 40% B; 10–11 min linear from 40 to 90% B; 11–12 min, 90% B; 12–13 min linear from 90 to 2% B; 13–18 min, 2% B. The separation of the temperature and injection volume was set at 50 °C and 2 µL, respectively. The analyses were performed in a positive ionization mode. The MS parameters were as follows: ion spray voltage, 4500 V; declustering potential, 30 V; entrance potential, 10 V; collision energy, 30 V (except from an argininosuccinic acid (Asa), cystathionine (Cth), L-cystine (Cys), L-homocystine (Hcy), δ-hydroxylysine (Hyl), L-lysine (Lys), and L-ornithine (Orn), 50 V) and collision cell exit potential, 50 V. Nitrogen was used as both a curtain gas and a collision gas. The temperature of an ion source, gas 1, and gas 2 was set at 600 °C, 60 psig, and 50 psig, respectively. The device operated in scheduled multiple reaction monitoring (sMRM) mode. The list of MRM transitions for 42 analytes and their corresponding internal standards is included in Table S1 in the Supplementary Materials.

### 2.5. Statistical Analysis

To perform statistical analyses, Statistica 10.0 (StatSoft Inc., Tulsa, OK, USA) and MetaboAnalyst 3.0 web platform (www.metaboanalyst.ca) were used [42]. A value of *p* < 0.05 was considered statistically significant. The data were analyzed by applying uni-and multivariate statistical tests. Firstly, the Shapiro-Wilk test was used to check the normality. The Mann-Whitney U test was used to compare variables without a normal distribution and the Levene’s test was applied to examine the equality of variances for variables with a normal distribution. When the Levene’s test result was not statistically significant (*p* > 0.05), which indicated the homogeneity of variance between groups, the Student’s *t*-test was performed. When the Levene’s test result was statistically significant (*p* < 0.05) Welch’s *t*-test was applied.

The multivariate statistical analyses consisted of a partial least squares discriminant analysis (PLS-DA) and a ROC curve analysis. Before using multivariate statistical analyses, the data were subjected to a process of normalization, transformation, and scaling. PLS-DA is a supervised multivariate analysis, in which after determining the number of factors needed to create a model, it is possible to assign samples to one of the two groups. PLS-DA also gives the opportunity to choose the variables most relevant to the classification of samples. To rank the AAs according to their importance in discrimination between groups, a variable importance in projection (VIP) score is frequently used. The higher the value of the VIP, the more important the variable is in the classification of patients. The ROC curves are used to assess the sensitivity and specificity of the discriminator. The greater the area under the curve (AUC), the better is the classification of the samples to one of the groups by the model. The ROC curves were created using the random forest algorithm and Monte Carlo cross-validation.

## 3. Results

### 3.1. Patients’ Characteristics

Thirty patients were enrolled: 13 in the asthmatic group and 17 in the control group. Control subjects were matched to the studied group in terms of age, sex, and ethnic origin. The characteristics of asthma patients and the control group are presented in Table 1.

### 3.2. Alterations in Serum-Free AA Profiles in Childhood Asthma

The applied methodology allowed a broad panel of free AA concentrations, including both proteinogenic and non-proteinogenic amino acids, to be measured in the studied serum samples. Thirty-five of 42 amino acids were detected and quantified in analyzed samples. The remaining seven amino acids (O-phospho-L-serine, O-phosphoethanolamine, L-homocitrulline, argininosuccinic acid, L-anserine, L-carnosine, L-homocysteine) occurred below the lower level of quantification in all analyzed samples. Some of the amino acids (δ-hydroxylysine, cystathionine, L-cystine) were detected only in part of the samples and were therefore excluded from further data analysis. Finally, the concentrations of the 32 amino acids were subjected to statistical analysis. The concentrations of AAs both in the asthmatic and control groups are shown in Table 2.

In order to evaluate differences in the serum metabolic profiles between asthmatic children and healthy subjects, univariate statistical analysis was used (Table 3). The applied tests demonstrated that the statistically significant differences between the studied groups occurred between the levels of the following five amino acids: taurine (Tau), ƴ-amino-n-butyric acid (GABA), DL-β-aminoisobutyric acid (bAib), L-arginine (Arg), and L-valine (Val). In asthmatics, the levels of taurine, L-valine, and DL-β-aminoisobutyric acid were reduced compared to the control group, while the levels of ƴ-amino-n-butyric acid and L-arginine were increased (Table 2).

### 3.3. Discrimination between Asthmatic Children And Healthy Subjects by AA Profiles

**Figure 1:**
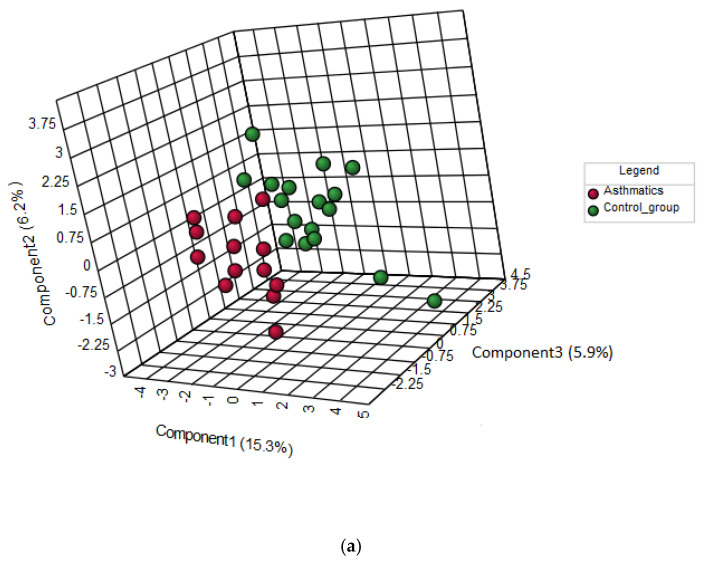

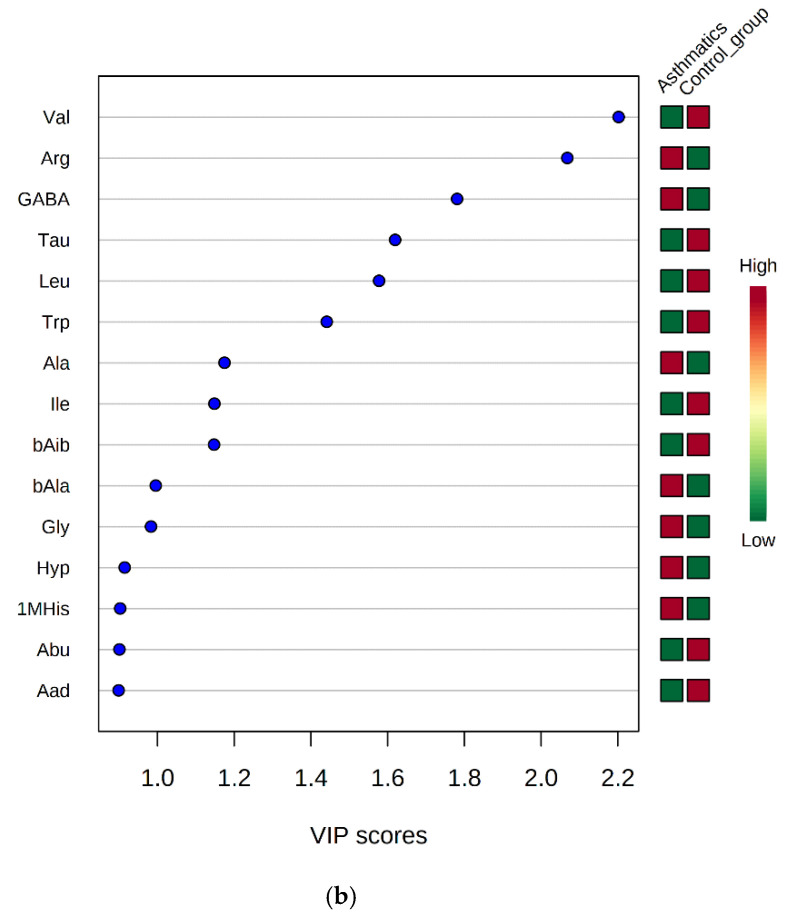
Results of a partial least squares discriminant analysis (PLS-DA) of free amino acid profiles determined in children with asthma and the control group. (**a**): Three-dimensional score plot (with explained variances shown in brackets). (**b**): The variables having the greatest importance in sample classification (amino acids with the highest variable importance in projection (VIP) score). Green and red boxes on the right hand side of the figure show if the levels of the respective amino acids are decreased or increased in the group.

To determine the differentiation ability of AA profiles, the multivariate statistical analysis was performed. A model for discrimination between two analyzed groups was created using PLS-DA analysis. Figure 1a is a diagram of distance vectors for the three components obtained by performing PLS-DA analysis (a score plot). The points corresponding to the samples belonging to the study group (asthmatics) have been marked in red, and the samples of people in the control group (healthy peers) have been marked by green. The figure shows a clear separation between the asthmatic children and controls. The PLS-DA analysis also revealed which variables have the greatest importance in the sample grouping. Figure 1b shows the variables listed according to their contribution in sample classification. The most differentiating AAs were as follows: taurine (Tau), L-valine (Val), L-arginine (Arg), ƴ-amino-n-butyric acid (GABA), L-leucine (Leu), and L-tryptophan (Trp).

**Figure 2:**
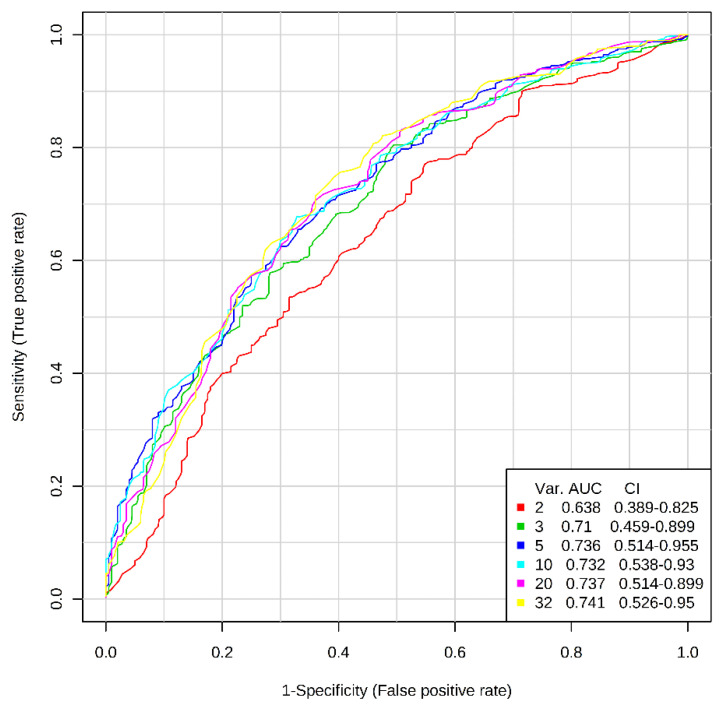
Receiver operating characteristics (ROC) curve analysis obtained for models based on different numbers of variables (serum levels of free amino acids), with area under the curve (AUC) values.

In the last step of statistical analysis, ROC curves were plotted for models, consisting of a different number of variables (32, 20, 10, 5, 3, 2) (Figure 2). It is noteworthy that increasing the number of AAs included in the model resulted in increasing the area under the ROC curves. The largest increase was observed when the number of variables was changed from two to five, while the addition of further variables into the model caused a slight increase in the area under the curve.

## 4. Discussion

In the face of the high prevalence of asthma in society and the need for the rapid implementation of treatment, it is important to have quantifiable indicators that correlate with the development of the disease. The aim of the study was to search for serum metabolic biomarkers among free AAs which may provide a distinction between asthmatic children and healthy ones. This paper applies the modern LC-MS/MS-based methodology that ensures the high specificity and sensitivity of the performed measurements and enables the simultaneous quantification of a broad AA profile (Table 2) in a small sample volume (40 μL).

Amino acids are considered to be mediators of immune activity in asthma and can act as antioxidants. In particular, amino acids such as glutamine, glutamate, glycine, and taurine have potentially protective effects, while others, like phenylalanine, may have adverse effects. Among the analyzed AAs, arginine deserves particular attention. Alterations in arginine homeostasis may lead to typical asthma symptoms, such as inflammation, airway hyperresponsiveness, and remodeling. Changes in arginine bioavailability may contribute to decreasing levels of bronchodilation NO and the excessive formation of peroxynitrite, which has procontractile properties [43,44].

**Figure 3:**
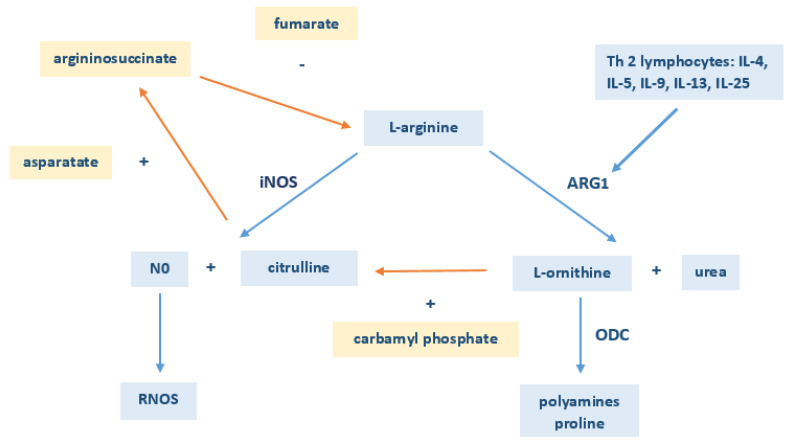
Metabolism of arginine in asthmatic children. The immature immune system in children manifested by decreased levels of T helper cells (Th 2) and inflammatory cytokines (IL-4, IL-5, IL-9, IL-13, IL-25) determines the decreased activity of arginase and the elevated level of arginine in the blood. Arginine cooperates with other metabolites within the network that affect each other’s concentrations. ARG1-arginase; iNOS-inducible nitric oxide synthase; RNOS-reactive oxygen species; ODC-ornithine decarboxylase.

In the available literature, there are conflicting reports on concentrations of this AA in both asthmatic children and adults, as well as information about the increased activity of arginase in asthmatics. Morris et al. [14] showed significantly lower levels of arginine in the serum of patients with asthma (45 ± 22 µM) compared to the control group (92 ± 29 µM). In turn, Lara et al. [12] and Fogarty et al. [13] did not confirm this association. In this study, the concentrations of arginine in the asthmatic children were significantly higher (110 ± 13 µM) than in healthy participants (93 ± 23 µM). It should be noted that the concentrations of this amino acid in both control groups analyzed by Morris et al. [14], and subjected to this study’s experiment, achieved a similar level. The different serum concentrations of arginine in asthmatic patients between this paper and Morris et al.’s study may result from a different age range of included individuals. Morris et al. [14] included patients aged from 2 to 52 years, while children from 3 to 18 years old participated in the present study. The increased concentration of arginine in asthmatic children may result from an immature immune system. Asthma is accompanied by the chronic inflammation of the airways. It is caused by immune cells, which include, inter alia, T helper cells. These cells are also directly involved in the metabolism of arginine, inducing the expression of the enzyme responsible for its distribution. Activated type 2 T helper cells (Th2) secrete cytokines, stimulating arginase activity [14,45]. Therefore, it may be assumed that the immature immune system in children determines the decreased activity of arginase, which is manifested by elevated levels of arginine in the blood (Figure 3). A recent study conducted by Kelly et al. [46] in a group of 411 three-year-old children provides strong evidence for dysregulated arginine metabolism in asthmatic children. Arginine levels in human serum depend on other metabolites within the network. It has been shown that arginine can interact with other metabolites and has joint influences on asthma. Arginine is produced from citrulline and acts as the substrate for nitric oxide (NO) synthesis, while its uptake can be inhibited by L-ornithine, L-lysine, and other metabolites involved in the pathways that regulate the levels of these metabolites (Figure 3). Kraj et al. [37] also revealed the altered metabolism of arginine in children with controlled asthma. The profile of AAs crucial for arginine metabolism significantly differed from that observed for acute asthma in adults and healthy individuals, although arginase activity remained unchanged when compared to the healthy group. Thus, effective disease control and the introduction of optimal treatment could have a significant impact on the maintenance of arginase enzymatic activity. Certainly, the dysregulated metabolism of arginine, its changing serum level, and its interaction with exogenous metabolites deserve further investigation as potential causative agents in asthma [45].

Another amino acid whose level was decreased in asthmatic children’s serum is taurine. It is a sulfur amino acid and it is not incorporated into proteins [47]. Taurine has a wide range of biological functions, serving, for example, as a cryoprotectant, antioxidant, and membrane stabilizer. Meanwhile, its deficiency leads to developmental abnormalities in the brain, heart, and skeletal muscles [47,48]. Taurine also plays a crucial role in the lungs [49]. It has been suggested to be an agonist of α4 subunit-containing GABAA receptors and may cause the relaxation of airway smooth muscle [50]. Comhair et al. [51] found a significantly higher level of taurine in asthmatic adults in the untargeted metabolomic study of plasma samples. The researchers suggested that the altered level of taurine is related to alterations in NO-associated transport [52]. The results obtained by Comhair et al. are contrary to the ones within this paper, which may have arisen from the age disparity of the studied populations (mean age 35 vs. 10 years). Zinellu et al. considered taurine as a potential biomarker of oxidative stress in asthma [53]. The mean concentration of plasma taurine was lower in asthmatics adults (mean age 58.8 ± 13.6 years, range 38–79) than in healthy ones, but this difference was not statistically significant. It is noteworthy that Zinellu et al.’s study focused exclusively on patients with mild stable asthma.

In this study, valine occurred at a decreased level in asthmatic children. Valine, one of the branched-chain amino acids (BCAA), is classified as an essential amino acid. BCAAs have many diverse functions in the human organism and they are crucial for energy, stress, and muscle metabolism (www.hmdb.ca). Valine was suggested as a biomarker of breast cancer [28,54], oral cancer [54], pancreatic cancer [54,55], liver disorders [56,57]⁠, colorectal adenoma [58], and type 2 diabetes [59], but there are not many publications about its role in asthma pathophysiology. Motta et al. [60] investigated the impact of different condensation temperatures on the exhaled breath condensate (EBC) metabolome in asthmatic adults. They demonstrated the decreased level of valine in the exhaled breath condensate of asthmatic patients (mean age 35 ± 1.2 years) compared to controls regardless of temperature. Researchers also suggested that the EBC metabolome depends on asthma severity. However, they did not profile metabolites in the serum/plasma of both adults and children suffering from asthma. Univariate and multivariate analyses conducted by Ghosh et al. [61] showed that serum levels of valine and 11 other metabolites (histidine, phenylalanine, lysine, asparagine, L-leucine, glutamate, lipid, isoleucine, N-acetylglycoproteins, citric acid, and glucose) were dysregulated in asthma–chronic obstructive pulmonary disease overlap (ACO) patients. This disease is similar to asthma but it is characterized by a faster decline in lung function, more frequent exacerbations, and a poorer quality of life when compared to asthma or chronic obstructive pulmonary disease alone [61]. Due to the lack of clear diagnostic and therapeutic guidelines, this may be an important finding.

Any discrepancies in the results between available studies are explainable. Metabolite profiles can vary depending upon multiple factors. A large number of results are not replicated due to the variety of biospecimens used in studies, like exhaled breath condensate, urine, plasma, and serum. Furthermore, there are not enough studies profiling metabolites in more than one biospecimen type, so it is not possible to determine the relationship between metabolites in different biological samples from the same person. Recently, Chiu et al. [62] simultaneously analyzed the metabolic profile of blood and urine related to IgE reactions for childhood asthma. Their research showed a significantly higher level of histidine in plasma and a lower concentration of 1-methylnicotinamide and trimethylamine N-oxide (TMAO) in the urine of asthmatic children compared to healthy individuals. Furthermore, Chiu et al. [62] correlated the plasma level of 3-hydroxybutyric acid, leucine, and valine to the urine concentration of hydroxy acids. However, this was a single comparative study, and more advanced research using different biospecimens in parallel, like exhaled breath condensate, urine, plasma, and serum, is needed. This issue could be one of the future directions in asthma metabolomic studies. Processing procedures and collection conditions can also affect the metabolome. Another important element that may account for the differences in study findings is the technique of metabolomic profiling. Nuclear magnetic resonance is a spectroscopic technique which uses the magnetic properties of atomic nuclei to generate structure information, and thus can identify metabolites in a studied biological fluid based on their unique chemical shift pattern and the intensity of the peak [63]. Meanwhile, tandem mass spectrometry combines chromatography, a technique used to separate metabolites, with MS, to measure their abundances. Research showed that measurements of NMR and MS may not always be comparable [10,15,63]. The results may also vary due to the heterogeneity of the asthma diagnostic criteria, with the variable use of medical diagnosis and/or spirometry criteria. In addition, it is known that the metabolome is very sensitive to external influences, including smoking, diet, and treatment regimen, and it changes with factors like BMI [19]. Certainly, more advanced studies are needed to identify all metabolic shifts caused by various environmental factors and physiological characteristics. In the nearest future, there will be a great need for standardization in the field of metabolomics and for the development of strict criteria for conducting and reporting metabolomic studies.

Among AAs with the highest discriminatory ability in childhood asthma, two derivatives of butyric acid, ƴ-amino-n-butyric acid (GABA) and DL-β-aminoisobutyric acid (bAib, BAIBA) were found. This is the first study that indicates alterations in the serum levels of these two metabolites in childhood asthma and asthma in general. In this study, the level of ƴ-amino-n-butyric acid was increased in asthmatic children compared to the control group, and the level of DL-β-aminoisobutyric acid was reduced. In the recent literature, there are findings that the GABAergic system exists in the airway epithelium and has a role in mucus overproduction in asthma [64]⁠. Meanwhile, DL-β-aminoisobutyric acid is involved in the regulation of carbohydrate and lipid metabolism and also decreases inflammatory reactions [65]. Thus, the occurrence of an inflammatory process in asthma may be associated with a reduced serum level of DL-β-aminoisobutyric acid. However, in the future, advanced and detailed studies are needed to clarify if the dysregulation of DL-β-aminoisobutyric acid production and/or its action is engaged in the pathogenesis of childhood asthma.

Due to the small size of the study groups in the performed AA profiling, various statistical analyses were used. It should be emphasized that both the uni- and multivariate statistics indicated the following AAs as the most differentiated: taurine, ƴ-amino-n-butyric acid, L-arginine, DL-β-aminoisobutyric acid, and L-valine. The altered concentration of these compounds can testify to their role in the pathogenesis of childhood asthma. Kelly et al. summarized the 21 metabolomic studies of adulthood and childhood asthma, all of which reported significant findings and concluded that individual metabolites and metabolic profiles measured in EBC, urine, plasma, and serum could identify people with asthma and even distinguish asthma phenotypes with a high discriminatory ability. Therefore, the introduction of the monitoring of the level of serum AAs in clinical practice is worth considering. This can be used both as a method of diagnostics and prognostics for controlling the course of therapy. Based on the plotted ROC curves, it can be assumed that a classification model is better if more variables are included. However, the inclusion of several of the most discriminative compounds is sufficient to carry out the classification. This is an important conclusion for the possible use of metabolomic analysis in the diagnosis of childhood asthma, considering that it will significantly reduce costs while maintaining adequate sensitivity and specificity.

## 5. Conclusions

The analysis of the human metabolic profile is a very promising tool for clinical applications since it is a sensitive indicator of both endogenous and exogenous factors affecting the patient’s body [66]. Changes in the human metabolome occur before changes in laboratory parameters and long before the onset of clinical symptoms. Due to the ability to measure these differences, metabolomics give hope for the detection of many diseases in the early stages of their development, which in turn can significantly increase the level of treatment effectiveness. Despite placing great hope in nitric oxide as a diagnostic marker for asthma in the past, today it can only be used to monitor treatment. Therefore, our results regarding the altered concentration of taurine, L-valine, DL-β-aminoisobutyric acid, ƴ-amino-n-butyric acid, and L-arginine in the serum of asthmatic children may contribute to the future introduction of new diagnostic markers. The involvement of AAs in the metabolic pathways proposed in this paper justifies the continuation of AA determination in various biospecimens collected from asthmatic patients. In the future, data on metabolomics in asthma should be integrated with the proteomic approach, as this may be the most informative integrative strategy.

## Figures and Tables

**Table 1:** Characteristics of the subjects.

Characteristics		Asthma Patients	Control Subjects
**No. of Subjects**		13	17
**Sex**			
Male		7 (53.8%)	11 (64.7%)
Female		6 (46.2%)	6 (35.3%)
**Age**			
Median		12	10
Range		4–16	3–18
**Asthma Severity**			
Mild		7 (53.8%)	
Moderate		4 (30.8%)	
Severe		2 (15.4%)	
**The Daily dose of Corticosteroids (Budesonide or Equivalent)**			
100–200 μg		6 (46.2%)	
250–350 μg		2 (15.4%)	
400–500 μg		3 (23.0%)	
>500 μg		1 (7.7%)	
Unknown		1 (7.7%)	
**Comorbidities**			
Hypoacusia		1	
Atopic Dermatitis		1	
Allergic Rhinitis		3	
Coeliac Disease		1	
Cholecystitis		1	
**Lung Function**			
FEV1/VC	Mean	85.25%	
	Range	67–94%	
FEV1	Mean	82.5%	
	Range	40–97%	
FVC EX	Mean	87.33%	
	Range	49–95%	
**Total IgE**			
Mean		278.61 kU/I	96.29 kU/I
Range		15.5–1035 kU/I	12.2–797 kU/I

**Table 2:** The determined concentrations of 32 amino acids in serum samples collected from children with asthma and the control group. Concentration values are given in µM.

Amino Acid	Abbreviation	Children with Asthma (*n* = 13)			Control Group (*n* = 17)		
		Median	Mean	SD	Median	Mean	SD
1-Methyl-L-Histidine	1MHis	3.09	4.42	4.4	1.74	1.93	1.73
3-Methyl-L-Histidine	3MHis	2.38	2.52	0.84	2.04	2.32	1.06
L-A-Aminoadipic Acid	Aad	0.64	0.66	0.2	0.66	0.78	0.29
L-A-Amino-N-Butyric Acid	Abu	15.56	16.72	5.24	18.98	19.53	6.16
L-Alanine	Ala	443.71	423.32	89.17	368.45	396.24	106.97
L-Arginine	Arg	112.59	109.97	12.66	87.67	93.15	22.77
L-Asparagine	Asn	46.87	49.32	5.23	51.71	52.24	11.23
L-Aspartic Acid	Asp	11.71	11.94	3.65	9.89	11.65	7.89
D,L-Β-Aminoisobutyric Acid	bAib	1.13	1.11	0.31	1.26	1.47	0.61
Β-Alanine	bAla	16.51	13.84	6.61	8.95	9.64	3.9
L-Citrulline	Cit	24.37	23.77	5.46	24.71	24.31	3.79
Ethanolamine	EtN	8.94	8.96	2.17	8.35	8.96	1.65
Ƴ-Amino-N-Butyric Acid	GABA	1.25	1.18	0.49	0.67	0.75	0.31
L-Glutamine	Gln	424.51	429.37	56.74	459.64	458.41	71.53
L-Glutamic Acid	Glu	52.97	58	19.77	64.58	62.66	18.27
Glycine	Gly	278.17	283.25	32.45	255.44	268.74	46.85
L-Histidine	His	69.11	68.38	7.93	61.62	66.83	13.1
Hydroxy-L-Proline	Hyp	22.02	22.44	14.17	13.32	15.94	6.71
L-Isoleucine	Ile	63.9	61.95	12.54	63.32	68.1	16.01
L-Leucine	Leu	92.42	97.89	18.21	104.96	111.25	25.25
L-Lysine	Lys	142.42	147.4	25.22	150.06	157.79	40.29
L-Methionine	Met	21.32	22.06	5.39	22.45	23.44	7.17
L-Ornithine	Orn	59.45	64.63	15.76	61.35	65.67	18.49
L-Phenylalanine	Phe	55.46	52.55	8.97	54.67	54.85	9.84
L-Proline	Pro	197.03	181.4	67.24	171.13	177.63	51.23
Sarcosine	Sar	1.28	1.24	0.74	1.26	1.26	0.51
L-Serine	Ser	133.87	140.51	14.25	140.57	139.22	22.81
Taurine	Tau	66.95	70.75	17.75	86.33	85.95	16.55
L-Threonine	Thr	94.75	101.29	27.81	97.24	96.91	20.15
L-Tryptophan	Trp	51.47	51.61	7.49	57.34	59.19	13.14
L-Tyrosine	Tyr	52.31	52.57	11.82	51.51	55.81	18.07
L-Valine	Val	165.8	173.07	29.58	195.68	204.36	38.34

**Table 3:** Results of univariate statistical analysis of serum-free amino acids in children with asthma and the control group. Bold type for *p* values indicates statistical significance.

Amino Acid	*p* Value					
	Shapiro–Wilk Test		Levene’s Test	Mann–Whitney U Test	Student’s *t*-Test	Welch’s *t*-Test
	Children with Asthma (*n* = 13)	Control Group (*n* = 17)				
**1MHis**	**0.052421**	**0.003404**		0.276532		
**3MHis**	0.875277	0.234623	0.327524		0.591304	
**Aad**	0.496739	0.112430	0.07644		0.216463	
**Abu**	0.621549	0.972163	0.644789		0.198044	
**Ala**	0.178613	0.371844	0.669371		0.467315	
**Arg**	0.288521	0.071223	**0.043268**			**0.016265**
**Asn**	0.469455	0.584648	**0.009499**			0.353783
**Asp**	0.753162	**0.000012**		0.241259		
**bAib**	0.694310	0.326845	**0.020441**			**0.045451**
**bAla**	0.385620	0.326401	**0.034083**			0.056388
**Cit**	0794372	0.951293	0.206713		0.753887	
**EtN**	0.128147	0.172044	0.617651		0.997393	
**GABA**	0.227097	0.609400	0.099223		**0.006383**	
**Gln**	0.389567	0.267469	0.442514		0.239702	
**Glu**	0.572162	0.911833	0.656593		0.509076	
**Gly**	0.437805	0.194367	0.324685		0.348727	
**His**	0.813939	0.357040	**0.015545**			0.690912
**Hyp**	0.093057	**0.017077**		0.167247		
**Ile**	0.738028	0.211595	0.309006		0.263792	
**Leu**	0.212462	0.279189	0.302909		0.118475	
**Lys**	0.752616	0.270535	0.367141		0.422490	
**Met**	0.792258	0.308677	0.289639		0.566910	
**Orn**	**0.026378**	0.288794		0.769551		
**Phe**	0.185401	0.541752	0.844958		0.515613	
**Pro**	0.173926	0.379691	0.219423		0.862817	
**Sar**	0.547304	0.953359	0.354722		0.953930	
**Ser**	0.132290	0.220079	0.300592		0.859334	
**Tau**	**0.011918**	0.966747		**0.012036**		
**Thr**	0.615151	0.330271	0.439175		0.619944	
**Trp**	0.301341	**0.046520**		0.131898		
**Tyr**	0.562556	0.220185	0.094828		0.580349	
**Val**	0.773498	0.170362	0.652018		**0.021465**	

## Supplementary Materials

The following are available online at https://www.mdpi.com/1660-4601/17/13/4758/s1, Table S1: MRM transitions for the analyzed amino acids (Q1 > Q3).
