# Supplementary material for: Alterations in Serum-Free Amino Acid Profiles in Childhood Asthma
Source: Int J Environ Res Public Health. 2020 Jul 2;17(13):4758. doi: 10.3390/ijerph17134758 (PMC7370195; doi:10.3390/ijerph17134758)
Supplement: Supplementary file 1 [file ijerph-17-04758-s001.pdf]

International Journal of
Environmental Research and Public Health

Table S1. MRM transitions for the analyzed amino acids (Q1>Q3).

Amino Acid	Analyte		Internal Standard	
	Q1 Mass (amu)	Q3 Mass (amu)	Q1 Mass (amu)	Q3 Mass (amu)
1-Methyl-L-Histidine	318.2	121.1	310.2	113.1
3-Methyl-L-Histidine	318.2	121.1	310.2	113.1
L-Alanine	238.2	121.1	230.2	113.1
L-Anserine	389.2	121.1	381.2	113.1
L-Arginine	323.2	121.1	315.2	113.1
Argininosuccinic Acid	439.2	121.1	431.2	113.1
L-Asparagine	281.2	121.1	273.2	113.1
L-Aspartic Acid	282.1	121.1	274.1	113.1
L-Carnosine	375.2	121.1	367.2	113.1
L-Citrulline	324.2	121.1	316.2	113.1
Cystathionine	519.3	121.1	503.3	113.1
L-Cystine	537.2	121.1	521.2	113.1
Ethanolamine	210.2	121.1	202.2	113.1
L-Glutamic Acid	296.2	121.1	288.2	113.1
L-Glutamine	295.2	121.1	287.2	113.1
Glycine	224.1	121.1	216.1	113.1
L-Histidine	304.2	121.1	296.2	113.1
L-Homocitrulline	338.2	121.1	330.2	113.1
L-Homocystine	565.3	121.1	549.3	113.1
Hydroxy-L-Proline	280.1	121.1	272.1	113.1
L-Isoleucine	280.2	121.1	272.2	113.1
L-Leucine	280.2	121.1	272.2	113.1
L-Lysine	443.3	121.1	427.3	113.1
L-Methionine	298.2	121.1	290.2	113.1
L-Ornithine	429.3	121.1	413.3	113.1
L-Phenylanalanine	314.2	121.1	306.2	113.1
O-Phosphoethanolamine	290.1	121.1	282.1	113.1
O-Phospho-L-Serine	334.1	121.1	326.1	113.1
L-Proline	264.2	121.1	256.2	113.1
Sarcosine	238.2	121.1	230.2	113.1
L-Serine	254.2	121.1	246.2	113.1
Taurine	274.1	121.1	266.1	113.1
L-Threonine	268.2	121.1	260.2	113.1
L-Tryptophan	353.2	121.1	345.2	113.1
L-Tyrosine	330.2	121.1	322.2	113.1
L-Valine	266.2	121.1	258.2	113.1
L-A-Aminoadipic Acid	310.2	121.1	302.2	113.1
L-A-Amino-N-Butyric Acid	252.2	121.1	244.2	113.1
B-Alanine	238.2	121.1	230.2	113.1
D,L-B-Aminoisobutyric Acid	252.2	121.1	244.2	113.1
Y-Amino-N-Butyric Acid	252.2	121.1	244.2	113.1
Δ-Hydroxylysine	459.3	121.1	443.3	113.1
